# Empirical evidence of organizational transformation: The subsequent consequence of the causal relationship between the adoption of circular economy strategies and their performance

**DOI:** 10.1016/j.heliyon.2024.e32987

**Published:** 2024-06-15

**Authors:** Llorenç Bagur-Femenías, Jordi Perramon, Maria del Mar Alonso-Almeida, Josep Llach

**Affiliations:** a UPF Barcelona School of Management, Barcelona, Spain; b Universidad Autónoma de Madrid, Madrid, Spain; c Universitat de Girona, Girona, Spain

**Keywords:** Circular economy, Performance, Organizational transformation, Strategy, Production

## Abstract

The adoption of circular economy (CE) strategies by companies—such as reduction, substitution, reuse, and others—is more necessary than ever to face recent challenges that have caused a rise in the price of raw materials, among other effects. However, incorporating CE strategies into the production process is not trivial because it can imply significant organizational transformation. To understand this transformation, this work analyses how the adoption of CE strategies impacts company performance and, consequently, the subsequent transformation of the company in adapting to this strategy. Based on a sample of 213 senior managers from companies in the manufacturing and service sectors, structural equation modelling is performed to contribute empirical evidence to the literature. The findings reveal that the adoption of CE strategies by companies can be more profitable than solely pursuing financial outcomes, as such adoption also impacts external stakeholders, society in general, and customers in particular. However, in return, the company must be open to deploying a certain degree of organizational transformation.

## 1 Introduction

One of the main objectives of the circular economy (CE) is the reduction in the amount of raw materials used for production, stemming from the principles that resources are scarce and that we should try to minimize their use and maximize their reuse, thus driving innovation [1,2]. The rise in the price of raw materials due to the worldwide conflicts that have occurred in recent years makes the use of CE strategies, such as reduction, substitution, and reuse, –concerning raw materials and other elements necessary for a company's production more necessary than ever. Thus, the incorporation of circular processes into the production chain could imply the transformation of those companies involved.

However, improving circularity and reducing the environmental impact of an organization are not concepts that perfectly overlap because circularity in an organization goes beyond better environmental performance [3]. The CE promotes industrial ecology, clean production and ecological modernization. Therefore, optimizing the use of resources allows a company to develop innovation and, thus, enjoy several benefits [2]. Nevertheless, according to the 2022 Circularity Gap Report, only 8.6 % of operations in the world are circular, despite the efforts of global international institutions and international agreements towards driving the CE. In addition, no advance was made between 2021 and 2022 in terms of circularity worldwide, with the exception of the abovementioned reduction strategy. Thus, when this kind of strategy is analysed in detail, it is found to contribute to a 28 % savings of raw materials.

Thereby, the reduction and recycling strategy are a good starting point for the transition towards circular production processes. For example, in a closed loop, waste becomes an input for processes, reducing both the need for raw materials in new production cycles and the generation of waste. Therefore, waste contributes to the circularity of companies and provides them with the possibility of transform, thus also providing them with a number of positive impacts. For this reason, a strategic reduction in the consumption of natural resources in a company's production processes and recycling could be considered a competitive advantage [2]. However, reduction, recycling or any other CE strategy requires changing how the company creates, delivers and captures value [4]. In addition, CE implementation requires the involvement of an organization's key stakeholders [1]. Thus, previous research empathize that reduction strategy has been incorporated into organizations like a strategic point to advance in the CE [2]. Other CE common strategies such as recycle also have been widely adopted by organizations (references) but it is not the case of other circular strategies like reuse [5].

The main pointed out reason is that CE strategies in practice are not free of multiple barriers -technological, market, institutional and cultural-among others [6,7]. In an effort to facilitate and push, CE in organizations, some voluntary international standards such as ISO 59000, or previous standards such as the French standard XP X30 901 or the British standard BS 8001 have been created [8]. Also, there is some evidence that Industry 4.0. Technologies pushed circular processes in organizations [[8], [9], [10]]. Nevertheless, on the one hand the internal uncertainty about the implementation, needed resources and change resistance [[10], [11], [12]] and on the other hand the uncertainty about the future legislation, lack of consumers trust and problems in the supply chain [[13], [14], [15]] have slowed down the development of the circular economy among organizations.

For this reason, several authors [2,6] asserted that circularity within firms continues in their infancy, despite the efforts of governments to push CE at all levels. Specifically, Dominko and colleagues [16] found that less than 15 % of those articles published on the CE up until 2021 are classified as involving firm economics, showing the scarce development of circular strategies in firms in practice. Nevertheless, most previous research has emphasized the positive impacts of circular strategy deployment on companies’ business and environmental performance [[17], [18], [19]] but with little practical evidence specially regarding organizational transformation [20].

Therefore, a number of researchers [3,6] have claimed that more research on the CE at the micro level is needed to analyse the transformation of companies when circular strategies are implemented in production processes.

Thus, there is room for improvement in many points of the supply chain for CE practice adoption [21]. Therefore, with the CE strategy in terms of operations in mind, this study explores its impact on performance and the transformation of the company derived from the use of such a strategy. Thus, this exploratory work seeks to shed light on circular processes’ impacts on business production and how these impacts drive organizational transformation. As Mouzas [22] asserted, “*It is well established in the literature that firms need to innovate and transform themselves, and our knowledge of what drives business transformation remains limited*”. Therefore, this paper aims to fill this gap.

To this end, this study makes a number of contributions to strategic management science. First, it sheds light on the impact of CE reduction strategies on company performance and main stakeholders. Second, it demonstrates how companies can transform their organization, structure and business both directly and indirectly due to circular strategy adoption. In fact, Sohal and De Vass [23] stated that understanding how a CE transforms a company is good for increasing circularity. Third, organizations need to have real references to evolve in terms of circular strategies that could drive so-called mimetic behaviour. Although the CE is a transformative system, it requires a global scope beyond some isolated organizations. Therefore, the contribution of this paper clarifies how circular processes impact corporate production and how these effects drive organizational change.

This paper contains, first, a literature review, an analysis model and a proposal of hypotheses. Second, the methodology and research sample are presented. Finally, the results, discussion and conclusions are presented.

## 2 Literature review

The adoption and implementation of CE strategies at the macro level support GDP growth, new jobs and economic sustainability in the long term [16]. Nevertheless, when the analysis is performed at the micro level, the results are mixed. In the following four subsections, the proposed study model is developed by analysing the circular economy reduction strategy and its impact on performance in a broad sense from an operations management perspective.

### 2.1 Circular economy strategy in production, business financial performance and organizational transformation

[17] asserted that “*the circular economy (CE) can bring not only benefits but also pitfalls to production processes, affecting a firm's economic performance*”.

Thus, several studies have found a positive relationship between CE practices and financial performance (for example [2,[24], [25], [26], [27]]). In fact, later research [28] confirmed that CE practices help organizations obtain better financial outcomes in terms of financial KPI improvement and total profitability. In the case of circular strategy reduction, internal savings due to energy, water and raw material reduction have been largely reported in organizations, even in complex situations [19].

In contrast, Gonçalves and colleagues [2] found that the link between CE and financial performance is weak and incipient. Similarly, D'Angelo and colleagues [17] reported the existence of an inverted U-shaped effect brought about by the number of CE strategies on economic performance. One of the reasons for this is that when a company focuses on multiple CE strategies, it could be perceived negatively by the market.

Therefore, CE strategies have been reported mostly as having a positive financial impact. However, the present authors advocate for more research, taking into account CE-specific strategies, among others, to strengthen the financial relevance of the CE in organizations.

Therefore, referring to previous research, the following hypothesis is proposed.

H1a A CE production strategy has a direct positive impact on business financial performance.

The adoption of CE strategies involves different activities and the reorganization of material and production flows [29] changes in business structure, manufacturing and supply chain processes; and new operations, routines and assets [10,30,31]. However, it is not free of additional challenges caused by the implementation of these new practices [[10], [11], [12],^32^].

Therefore, CE strategies allow for the capitalization of new streams of savings and revenue [20]. Nevertheless, Gonçalves and colleagues [2] asserted that the lack of financial incentives could hinder the evolution of circular operations within companies; these authors revised the previous research linking the circular economy and financial performance and, through a bibliometric and content analysis, found that it is important to specifically study the gains in financial operations and changes in the company and business. Given that financial constraints are one of the main obstacles to the implementation of the CE, savings in production processes can be invested in increasing circular business transformation. Circular transformation includes the deployment of other circular strategies, such as reuse, repair, and refurbishment [5], driving organizational growth, creating new departments and services, and consequently changing the organization [31].

In fact, Sohal and De Vass [23] explained that circular strategies have the potential to produce novel financial models that contribute to transforming organizations. Such organizational transformation can act as a virtuous cycle driving a company's revenue, cost reduction and profit [33], which are financial resources that are needed for further circular strategy implementation.

Thus, referring to previous research, the following hypothesis is proposed.

H1b A CE production strategy has a positive impact on organizational transformation through organizational financial performance.

### 2.2 Circular economy strategy in production, environmental and social performance and organizational transformation

Previous research has asserted that CE strategies have a positive impact on environmental and social performance [18].

Today, climate change, air pollution, the depletion of natural resources, desertification and the loss of diversity are some of the current environmental challenges that humans must face. Thus, activists and scientists advocate for the urgency in deploying specific CE strategies in companies to make a real contribution to planet sustainability [34]. Previous research has shown that CE reduction strategies have a positive impact on the planet due to fewer natural resources being used for production [18,19].

In fact, Moraga and colleagues [35] asserted that the aim of circularity in organizations should be to create circular loops to maintain the value of materials and energy as long as possible, with production and consumption systems reducing the total consumption of raw materials and having a positive impact on environmental performance. In line with this reasoning, green procurement implementation can be *“an opportunity to achieve a faster transition from a linear to a circular economic system*” [1]. Therefore, CE strategy could lead to a change in supply chain procurement with the goal of reducing total inputs in the production process.

Finally, the goal of the CE strategy is to minimize overall material consumption in the system by increasing efficiency both directly in terms of production and indirectly in terms of consumption, producing more sustainable products and services [36,37].

Regarding social performance, Silvestri and colleagues [38] asserted that CE strategies allow businesses to contribute to achieving the sustainable goals of the 2030 agenda and pushing for more conscious consumption and sustainability [39]. Thus, CE strategy could have a direct effect on SDG 9 industry, innovation and infrastructure; SDG 12 responsible consumption and production; and SDG 13 climate action [40]. In addition, several researchers have emphasized the positive links among CE strategy adoption, job creation and social wealth [41].

Therefore, based on previous research, the following hypothesis is proposed.

H2a A CE production strategy has a direct positive impact on environmental and social performance.

The positive results obtained on environment and social performance can act as a virtuous circle and produce the greatest awareness of the importance of CE strategy adoption. In addition, an improvement in business efficiency and contribution to the community can improve the brand and reputation of companies in the market [42,43].

Therefore, companies could send a signal to the market about their real engagement with sustainability [1]. This situation boosts circular transformation in organizations. Prosman and Cagliano [5] asserted that the main changes include activities related to other more advanced circular strategies or the repurposing of the businesses of the company, which can include the offering of new products and services or the creation of new departments [30,31].

Therefore, the following hypothesis is proposed.

H2b A CE production strategy has a positive impact on organizational transformation through environmental and social performance.

### 2.3 Circular economy strategy in production, customer performance and organizational transformation

Collaboration among actors has also been reported to be a key enabler for the efficient use of resources, such as water or raw materials [1,44]. In particular, collaboration with customers could push the consumption of water or energy and other raw materials in production, thus improving the global resource efficiency of the company [45]. However, the most important impact is the possibility that this collaboration could provide for the extension of business competitiveness by satisfying new customer needs and requirements and helping find new markets. Therefore, customer involvement is crucial in the business transition to a circular economy [3].

Nevertheless, not all types of customers are ready to understand circular practices in businesses or operations. On the one hand, previous research has widely emphasized that these circular attributes in products and/or businesses add value for the customer and could drive brand loyalty [3]. Therefore, the above authors recommended making the circular attributes of products or services visible.

Therefore, the circular attributes in a product or production process should be highlighted so that customers can clearly identify their benefits, which could increase their positive value in customers’ minds [39]. Moreover, some consumers concerned about climate change and the planet could specifically seek out this type of product [16]. Thus, circular strategies in production provide an opportunity to access a new segment of customers and drive the company to create new products and services [33].

On the other hand, some research has advised that the presence of circular strategies could negatively influence consumers’ attitudes towards a product [46]. The main reason for this is that some customers could perceive lower quality or risk in circular products [3]. Thus, although mixed results have been presented by previous research, mostly positive impacts have been reported. Therefore, the following hypothesis is proposed.

H3a A CE reduction production strategy has a positive impact on customer performance.

A company can increase the value of its products and services by improving their quality, functionality and/or aesthetics by means of replacing components and repairing parts on products to increase their life and circularity [47]. Knäble and colleagues [17] emphasized the central role of customers in circularity adoption among companies. Specifically, the monitoring of customers’ perceptions regarding CE implementation and its acceptance is crucial to secure the success of such practices.

Nevertheless, as Sohal and De Vass [23] noted, the changes driven by circular strategies may cause organizations to rethink their value proposition and redesign their organizational structure. Thus, this strategy implies that a company needs some transformation with a customer focus [48], providing value added to customers to avoid them perceiving that CE strategies divert other aspects of the company, such as production quality or customer care [17]. Therefore, the following hypothesis is proposed.

H3b A CE reduction production strategy has a positive impact on organizational transformation through customer performance.

### 2.4 Circular economy practices in production, employees’ capabilities and organizational transformation

Human resources are critical in all types of organizations. Nevertheless, they are one of the main obstacles to organizational change. In this sense, previous research has reported that among the obstacles to adopting the CE in production are the lack of internal knowledge and resistance to change [7].

For this reason, Negra and colleagues [49] asserted that employees need to acquire new skills and capabilities to facilitate CE deployment in organizations. These skills and capabilities should include technical and personal issues. Especially, when advance technologies are included in the industrial processes [8]. Among other factors, flexibility and specific knowledge are relevant to deploying CE strategies. Therefore, the following hypothesis is proposed.

H4a A CE reduction production strategy has a direct positive impact on employees' capabilities.

The acquisition of CE knowledge, skills and capabilities is crucial because employees must participate in the discussion of the procedures and routines to transform production processes and achieve the necessary new services and processes to develop this approach [50]. This is especially relevant when circular strategies are seen as not merely a fad but, rather, a long-term organizational commitment. Nevertheless, CE transformation is not easy [51]. Therefore, it is important for employees to understand the potential benefits for the company, customers and themselves in the long term [52].

Thus, when human resources increase their qualifications, business transformation is much easier because interdisciplinary expert teams can create new and interrelated knowledge to transform the organization [7] and make better use of resources [53]. In addition, human resources influence the purpose of the organization and contribute to its environmental adaptation, which could lead to a competitive advantage [54], a specific set of organizational capabilities [48] and organizational transformation. Therefore, the following hypothesis is proposed.

H4b A CE reduction production strategy has a positive impact on organizational transformation through employees' capabilities.

## 3 Methodology

### 3.1 Data and method

A structured questionnaire was administered to senior managers (with an average experience of over 20 years in their positions at their respective firms) during 2021 to test the previously proposed hypotheses. There were two sections in the questionnaire. The first section included questions related to respondents' profiles. Table 1 presents the descriptive statistics of respondents. The second section included questions related to respondents’ perceptions of the use of CE strategies and their impact on performance. For all the variables, respondents were required to choose a value from a 5-point Likert scale (with 1 meaning “totally disagree” and 5 meaning “totally agree”).

**Table 1:** Descriptive statistics of the respondents.

Gender	n	%	Education level	n	%	Sector	n	%	Position	n	%
**Male**	97	45.5	**Grade**	40	18.8	**Industry**	47	22.1	**Administrative officer**	33	15.5
**Female**	116	55.5	**Master**	173	81.2	**Services**	94	44.1	**Executive**	84	39.4
						**Others**	72	33.8	**Technician**	27	31.5
									**Others**	29	13.6
**Total**	213	100 %		213	100 %		213	100 %		213	100 %

### 3.2 Measures

Based on the previous literature and in light of the proposed hypotheses, six dimensions were explored. The first dimension was circular economy strategy (CES). The variables included to measure this strategy to reduce the consumption of natural resources in production were ‘raw material savings’ and ‘water and other energy savings’. The second dimension, business financial performance (FP), included four variables: ‘income increase’, ‘cost reduction’, ‘profit increase’ and ‘financial KPI improvement’. Environmental and social performance (ESP) was measured in terms of six dimensions: ‘sustainable development improvement’, ‘green purchase development’, ‘delayed climate change’, ‘maintaining the planet for future generations’ and ‘contribution to the fulfilment of the 2030 agenda’. Next, customer performance (CP) was composed of three variables: ‘access to new customer segments’, ‘increased customer satisfaction’ and ‘increased customer loyalty’. To measure employees' capabilities (EC) due to the introduction of the CE, the following two variables were used: ‘greater need for training courses, especially in the field of the circular economy’ and ‘increased level of professional qualification’. Finally, the variables used to measure organizational transformation (OT) were ‘create a technical repair department in the company’, ‘use products that can be utilized for more than one function’ and ‘use products whose elements can be easily disassembled and readapted for later use’. The Appendix details the operationalization of the individual variables with their sources.

### 3.3 Method

To test the causal model of this study, Structural Equation Modelling (SEM) was performed. SEM has become one of most popular statistical techniques in the last years in the social science field. According to Rahman and colleague [55] “*this technique has got popularity because of the sophistication of its underlying theory and its potential for addressing important substantive questions*” like the hypothesis above raised. The main characteristic of SEM that has capture of the attention of the academia is its very potential of handling complex relationships among variables, where some variables can be hypothetical or unobserved. The two main typologies of SEM coexist in the literature, covariance-based SEM (CB-SEM) and variance-based partial least squares SEM (PLS-SEM) with different approaches and assumptions.

In the case of the present study, since the sample size was over 100 and the latent dimensions of the causal model were reflective, the model was tested with the CB-SEM approach, using the maximum likelihood method, following the recommendations of [56].

## 4 Results

The results are detailed in two subsections following the statistical process carried out. The first subsection presents the validity and reliability of the measurement model. Next, the proposed working model is tested using the maximum likelihood method and EQS (version 6.4).

### 4.1 Measurement model

Table 2 presents the reliability of the validity attributes of the reflective first-order dimensions and their individual variables. First, individual variable reliability was assessed by item standardized loadings. All the items were over the cutoff of 0.707, as proposed by Carmines and Zeller [57], except two variables concerning the financial performance dimension. However, the authors decided to maintain these items because their importance and high standardized loadings were very close to the minimum criterion.

**Table 2:** Reflective first-order measurement model: reliability and convergent validity.

	Circular economy strategy		Financial performance		Environmental and social performance		Customer performance		Employees' capabilities		Organizational transformation	
	code	load	code	load	code	load	code	load	code	load	code	load
	**CES1**	0.949	**FP1**	0.693	**ES1**	0.816	**CP1**	0.811	**EP1**	0.898	**OT1**	0.799
	**CES2**	0.949	**FP2**	0.688	**ES2**	0.810	**CP2**	0.874	**EP2**	0.898	**OT2**	0.912
			**FP3**	0.781	**ES3**	0.863	**CP3**	0.826			**OT3**	0.912
			**FP4**	0.806	**ES4**	0.860						
					**ES5**	0.888						
					**ES6**	0.841						
**C's α**^a^	0.890		0.715		0.920		0.782		0.758		0.829	
**CR**^b^	0.948		0.831		0.938		0.876		0.893		0.908	
**AVE**^a^	0.901		0.533		0.717		0.701		0.806		0.767	

***Average Variance Extracted.

a *Cronbach's alpha*.

b *Composite reliability*.

The internal consistency reliability of the dimensions was assessed using Cronbach's alpha and composite reliability. In both cases, all the dimensions had values over 0.7, which is the minimum internal criterion proposed by Nunally [58] that confers reliability to the measures. Finally, convergent validity was assessed using average variance extracted (AVE), which proves the unidimensionality of the latent variables. Again, the values of all the latent variables were over the minimum criterion of 0.5 [59].

Table 3 proves the discriminant validity of the dimensions with a comparison of the square root of the AVE and the correlation between dimensions. Since the square root of the AVE is always higher than the interdimension correlations, it can be confirmed that each factor represents a separate dimension [60].

**Table 3:** Correlation matrix and discriminant validity.

	1	2	3	4	5	6
**CES**	0.949^a^					
**FP**	0.034*	0.744				
**ESP**	0.257**	0.150**	0.847			
**CP**	0.187**	0.032*	0.425**	0.837		
**EP**	0.261**	0.187**	0.311**	0.501**	0.898	
**OT**	0.471**	0.199**	0.169**	0.227**	0.372**	0.876

*significant at p value < 0.05; ** significant at p value < 0.01.

a Square root of AVE in the diagonal.

### 4.2 Structural model

To test the structural model, structural equation modelling (SEM) was performed using the maximum likelihood method. Table 4 presents the overall fitness indices obtained. According to Fornell and Larcker [61], who stated that at least three indices are required for valid compilation, the explanatory power of the model was confirmed.

**Table 4:** Indices tested for the model fit.

Assessment item	Values	Recommended value	Source
X2/df (normed chi-squared)	4.508	2 < x < 5	[62]
BBNFI (Bentler Bonnet Non-Normed Fit Index)	0.861	>0.9	[[63], [64], [65]]
BB Normed Fit Index	0.855	>0.8	
CFI (comparative fit index)	0.800	>0.8	
RMSEA (root mean square error of approx.)	0.097	<0.1	[65]

**Fig. 1:**
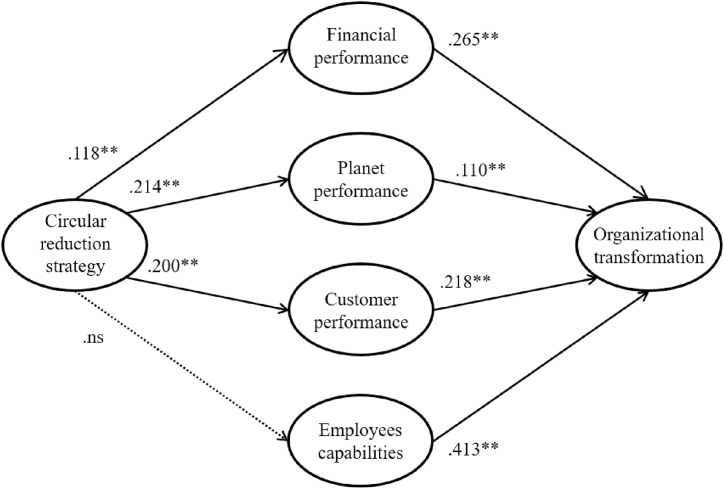
Standardized solution of the working model. **robust statistics significant at the 5 % level. ns: not significant relationship.

Fig. 1 presents the standardized solution to the proposed model. Examining the results for specific hypotheses, it can be observed that all of the hypotheses, except for that concerning the relationship between circular reduction strategy and employees’ capabilities, are supported at the 0.05 level.

## 5 Discussion

Several previous studies have focused on demonstrating the positive impact of CE strategies on different economic and organizational performance dimensions. However, less research has addressed how specific CE production strategies contribute to the different dimensions of organizational performance and transformation. Although the results of this study confirm the previous literature, these findings also provide new insights.

Thus, the impact of CE strategies is different depending on the strategy being implemented. Previous research asserted that some strategies are easier to promote than are others [17]. Therefore, the prioritization of CE strategies with higher acceptance or easy implementation is needed when trying to identify the largest impact.

Thus, the CE reduction strategy of the firm has a positive direct impact on financial, environmental and customer performance but not on employees’ capabilities. Therefore, H1a, H2a and H3a are supported, but H4a is rejected.

Among the supported hypotheses, the findings show that the greatest impact of a CE strategy is on environmental performance (H2a). This finding is in line with the overarching objective of the CE—to try to provide a better planet for future generations—and is aligned with SDG [34,40]. Moreover, the goal of improving environmental performance drives changes in the supply chain, such as green procurement, which extends the improvement of the environment beyond the limits of an individual company. This finding reinforces the importance to the extend the circular processes beyond the limits of the organization.

In fact, the second greatest impact of a CE strategy is associated with customer performance (H3a). Consistent with previous research, circular economy practices can contribute to satisfying the new needs and requirements of customers and the possibility of reaching new markets concerned with the environment [3,16]. Consumers are increasingly seeking healthier and more sustainable products and services as a way to make a small contribution to the planet and promote more sustainable consumption that contributes to improving their own quality of life [16]. Therefore, the diffusion and promotion of its circular practices can improve a company's brand and image in consumers' eyes.

Finally, CE strategy has a positive impact on financial performance (H1a) but to a lesser extent than that on environmental and customer performance. This finding confirms previous research regarding the improvement of financial figures in general, although the specific numbers should be analysed in more detail.

Therefore, our findings (H1a, H2a and H3a) confirm the positive impact of CE reduction strategy implementation on companies and provide a basis on which to encourage them to evolve to a circular organization, beyond those laws pushing the CE.

Nevertheless, the implementation of the CE reduction strategy has not resulted in an increase in employees’ capabilities (H4a). Thus, our results do not support the need for specific training and a higher level of professional qualification derived from the CE reduction strategy. Usually, as reported in previous research, changes in technology, production or processes involve the need for new skills and capabilities for employees and, as a consequence, an increase in the level of professional qualification. Nevertheless, this finding shows that the transition to a new production model does not always involve changes in human resource capabilities.

The increase in their level of skills and capabilities is linked with employees' level of involvement in production processes and the degree of automatization. A circular reduction strategy does not always require a high level of employee involvement. This finding can also encourage companies to evolve towards the circular economy because it proves that this process can be easier and less expensive than they had imagined. Moreover, this finding shows that some CE strategies, such as reduction strategies, could be deployed in organizations without resistance or large investments because they do not require changes in the workplace or employees’ capabilities.

With regard to business transformation, according to the results, all the causal relationships between CE strategy performance and organizational transformation are positive and significant. Nevertheless, considering this evidence and the previous result that there is not a significant relationship between circular reduction strategy and employees’ capabilities, H1b, H2b and H3b are supported, but H4b is rejected.

However, it is noteworthy that the highest impact on organizational transformation comes from employees' capabilities, as mentioned above (H4b). This finding reinforces the idea that circular strategies contribute to organizational transformation, but depending on the CE strategy adopted, the impact among human resources is not as heavy as has been suggested by previous research [7]. In contrast, circular strategies could provide human resource opportunities in organizations and serve as a source of organizational engagement because those changes could be perceived as good for both the company and the planet. Another possible explanation is that organizations with human resources who are more informed about the circular economy paradigm are able to transform the organization without changing employees’ capabilities derived from their own previous knowledge.

In addition, employees are the main resources that make organizational changes possible. Therefore, it seems that circular strategies could be a driver of organizational transformation supported by employees. Thus, knowledge can be a driver of organizational transformation, although in the beginning of such transformation, there is no perception of the improvement of employees’ capabilities. Obviously, new knowledge and skills that are more complex may be needed for some circular strategies but not for simple strategies, such as reduction.

With regard to the impact of financial performance on organizational transformation, it is accepted that a CE production strategy has a positive impact on organizational transformation, mediated by business financial performance (H1b). This finding is consistent with previous research and confirms that savings in production processes due to a CE reduction strategy can be used to implement another circular strategy that pushes organizational transformation itself. In fact, CE reduction strategies could provide excess cash that is useful for mobilizing resources to push organizational change [33]. Thus, the mobilization of resources allows for the development of experiments and for the recombination of old and new practices to allow employees to familiarize themselves with the new methods and processes and contribute to developing growth strategies [1].

In the case of customer performance, it is confirmed that a CE production strategy has a positive impact on organizational transformation, mediated by customer performance (H3b). Thus, this finding shows that organizational change is needed to properly respond to new requirements and potential new customers. Therefore, this finding corroborates that current and potential customers are an active force leading organizational transformation but that this force depends on the circular strategy being implemented. When the strategies are more visible to customers, they have more power in organizational transformation. Nevertheless [17], advised that customers are key not only in CE strategy adoption but also in assessing which strategy has been adequately adopted and which has not.

Finally, it is also confirmed that a CE reduction production strategy has a positive impact on organizational transformation, mediated by environmental and social performance (H2b). Environmental and social performance are relevant to organizational transformation, especially in terms of their role in accounting for stakeholders and the value of the circular economy in terms of protecting the planet. Thus, our findings reinforce the role of circular strategies in organizations as enhancers through which to achieve higher external environmental and social objectives, such as SDG [40].

In summary, these findings suggest that the implementation of circular reduction strategies may have a direct and indirect impact on organizational transformation through financial, planet and customer performance and that although not necessary, the highest impact on organizational transformation is the result of higher employee capabilities. In the transition to a circular economy, organizational transformation can be accomplished to any degree depending on the type of circular strategy adopted by the company, the type of human resources that it has and the capacity to face consumers’ requirements and future needs.

## 6 Conclusions

There is no doubt that the competitive environment for firms is changing rapidly. The rise in commodity prices in recent years, due to market instability, is just one more piece of evidence of this ongoing change. Therefore, through the implementation of the circular economy, companies can contribute to reducing operational costs to maintain their competitiveness in addition to the need to meet UNESCO's sustainable development objectives.

Nevertheless, the literature that has analysed the impact of the implementation of these activities on business transformation is still scarce [3]. To this end, this paper attempts to shed light on the relationships among the implementation of a CE cost reduction strategy, the company's performance and the subsequent transformation of the company to adapt to this strategy. As a result of this research work, a number of academic, practical and policy-making conclusions have emerged. Additionally, some limitations and future research have been noted.

First, regarding the impact of CE reduction strategy on company performance and the main stakeholders, this research finds that multiple positive impacts for the main internal and external stakeholders can be achieved with simple circular strategies, even in complex environments such as the current one. This finding is in line with Sohal and De Vass [23] assertion that circular strategy adoption can be more profitable than can just pursuing financial outcomes. Also, the power of voluntary international standards adoption or technologies of industry 4.0 should be taken into account [8].

Second, this study demonstrates how companies can transform their organization, structure, business model and growth strategies through the adoption of circular strategies. Thus, those findings reinforce that circular strategies contribute to the redesign of the business model, production processes and organizational value, which push organizational transformation. This organizational transformation could have different implications, shapes and scopes. Consequently, more qualitative and quantitative research on this topic is needed. In addition, other dimensions such as previously mentioned should be included in the analysis.

Third, organizations need to have real references that can drive so-called mimetic behaviour to evolve into circular strategies. These findings show that circular strategies can be implemented for all types of companies, even small companies. Thus, it is important to note that it is not necessary for a company to adopt very complex circular strategies to achieve positive impacts and that the organization should be open to deploying a certain degree of transformation. Therefore, a starting point based on a reduction strategy seems to be the easiest and best way to transition towards a circular economy, even for small companies. However, this could be a first step towards implementing other circular strategies and increasing circularity in organizations, not just an excuse to continue operating in terms of linear production [66]. Thus, the adoption of voluntary international standards can contribute to the importance of systematising management to advance in the processes’ circularity [8].

For academics, the results suggest that the implementation of a circular reduction strategy has deep implications for strategic and operations management, production and operation processes and organizational behaviour. Thus, research on these fields considering the circular economy view is relevant for companies’ competitiveness and responses to the changing environment. Specifically, this applies to the current necessity to reduce the use of raw materials and other natural resources.

For managers, the results show that operational cost reduction efforts have an impact on both profit and loss. An improvement not only for the planet but also, beyond that, in terms of customer satisfaction and financial results, as Testa and colleague [3] pointed out, is found. Therefore, financial investments in the optimization of production processes are beneficial in the short and long term, at both the macro and micro levels. Organizational transformation is necessary for firms to achieve the higher-level benefits of circular strategies. In addition, these changes could facilitate the transition to other circular strategies and increased organizational transformation.

Finally, policymakers can play a leading role as diffusers of circular economy benefits and initiatives that are more suitable for organizations depending on their location [39]. Previous research has shown the positive impact of a balance between coercive and normative pressures to contribute to expanding circular economy adoption. Another potential initiative to push the adoption of circularity in organizations is offering low-tax schemes for circular production and products [39].

The present work has several limitations. The first limitation is common to most surveys of this type. Collecting data by surveying one manager per company may involve elements of subjectivity. However, this possible bias must be countered by a large number of surveys being conducted, as confirmed by the results of statistical methods. Second, these results are not generalizable because they focus on a specific country. It would therefore be necessary to continue this line of research in other geographical areas to understand potential differences across countries.

Finally, these results open up future lines of research. On the one hand, it would be interesting to analyse the results according to the sectors in which the companies operate. A cost reduction strategy should have a different impact on business performance, particularly financial performance, when the company is highly automated or labour intensive. Additionally, the role of consumers in the advancement of the circular economy needs more research. Similarly, the results obtained in this paper need to be contrasted with those for other geographical areas. Furthermore, the influence of governmental policies to push the circular economy at all levels could to some extent influence the degree of adoption of this model.

## Data availability statement

The authors do not have permission to share data.

## CRediT authorship contribution statement

**Llorenç Bagur-Femenías:** Writing – review & editing, Writing – original draft, Conceptualization. **Jordi Perramon:** Writing – review & editing, Writing – original draft, Conceptualization. **Maria del Mar Alonso-Almeida:** Writing – review & editing, Writing – original draft, Supervision, Investigation, Formal analysis, Conceptualization. **Josep Llach:** Writing – review & editing, Writing – original draft, Methodology, Investigation, Formal analysis, Data curation, Conceptualization.

## Declaration of competing interest

The authors declare that they have no known competing financial interests or personal relationships that could have appeared to influence the work reported in this paper.

## Figures and Tables

**Table:** 

Dimension	Code	Variable	Mean	St. Dev.	Source
**Circular reduction strategy**	**CRS1**	Raw materials savings	3.58	1.24	[1,19,34]
	**CRS2**	Water and other energy savings	4.01	1.18	
**Financial performance**	**FP1**	Income increase	3.52	0.86	[2,[24], [25], [26], [27]]
	**FP2**	Cost reduction	4.12	0.95	
	**FP3**	Profit increase	3.95	0.73	
	**FP4**	Financial KPI improvement	3.59	0.81	
**Planet performance**	**PP1**	Sustainable development improvement	4.43	0.63	[1,16,19]
	**PP2**	Green purchase development	4.34	0.68	
	**PP3**	Delay climate change	4.48	0.62	
	**PP4**	Maintaining the planet for future generations	4.52	0.62	
	**PP5**	Contribution to the fulfilment of the 2030 Agenda	4.46	0.61	
	**PP6**	Promote more sustainable consumption	4.44	0.64	
**Customer performance**	**CP1**	Access to new customers segments	4.68	0.46	[1,3,46]
	**CP2**	Increase customer satisfaction	4.07	0.74	
	**CP3**	Increase customer loyalty	3.99	0.81	
**Employees capabilities**	**EC1**	Greater need for training courses, especially in the field of circular economy	4.27	0.66	[49]
	**EC2**	Increase the level of professional qualification	4.04	0.76	
**Organizational transformation**	**OT1**	Create a technical repairs department in the company	3.52	1.07	[7,47]
	**OT2**	Use products that can be utilized for more than one function	3.89	0.80	
	**OT3**	Use products whose elements can be easily disassembled and readapted for later use	4.02	0.83	
